# Dengue Outbreak — Federated States of Micronesia, 2012–2013

**Published:** 2013-07-19

**Authors:** Livinston A. Taulung, Carolee Masao, Hibson Palik, Marcus Samo, Lisa Barrow, Moses Pretrick, Eric J. Nilles, Jacob Kool, Fred Aure, Russell Simmons, Frederick Moore, Alyssa Pyke, Carmel Taylor, Van-Mai Cao-Lormeau, Tyler M. Sharp, Tai-Ho Chen

**Affiliations:** Kosrae State Epinet Team members, Kosrae; Dept of Health and Social Affairs, Federated States of Micronesia; Div of Pacific Technical Support, World Health Organization, Suva, Fiji; Research Institute of Tropical Medicine, Muntinlupa City, Philippines; Queensland Health Forensic and Scientific Svcs, Brisbane, Australia; Institut Louis Malarde, Papeete, French Polynesia; Div of Vector-Borne Disease, San Juan, Puerto Rico; Div of Global Migration and Quarantine, Honolulu, Hawaii, CDC

On September 26, 2012, a woman aged 35 years from Kosrae in the Federated States of Micronesia (FSM) was hospitalized with fever, headache, muscle pain, vomiting, leukopenia, and thrombocytopenia. A rapid diagnostic test (RDT) (Dengue Duo, Standard Diagnostics Inc.) was positive for dengue virus (DENV) nonstructural protein-1 (NS1). During the next week, seven more persons with suspected dengue were tested with the RDT, of whom three were RDT-positive for NS1 or anti-DENV immunoglobulin M (IgM). During October, the Kosrae State Department of Health Services, with support from the FSM Department of Health and Social Affairs and the World Health Organization (WHO), responded to the outbreak with enhanced surveillance, training in clinical management, analysis of hospital surge capacity, a rapid mosquito survey to identify species and distributions, and control measures. By March 14, 2013, approximately 3.7% of Kosrae State residents had been hospitalized with suspected dengue. The outbreak consumed scarce medical and public health services, including outpatient, inpatient, and laboratory services, resulting in redirection of human and material resources from other important medical and public health activities. Because the health consequences of dengue can be substantial in resource-limited settings, Pacific Island nations might wish to consider preparedness measures for dengue outbreaks such as developing and testing outbreak response plans and ensuring adequate capacity for epidemiologic surveillance and laboratory testing.

## Investigation and Results

Kosrae, with a population of 6,600, is a small (42 square miles [109 square kilometers]) volcanic island that forms most of the land mass of Kosrae State, one of the four states of FSM. Kosrae has four municipalities, of which Lelu is the administrative center and home of 33% of the state’s residents and the 40-bed Kosrae State Hospital. The only previously documented DENV transmission on Kosrae was an outbreak of dengue virus serotype 2 (DENV-2) in 1998 (1).

During October 2012, the number of dengue cases continued to increase, and in late October an epidemiologist from WHO was deployed to provide technical assistance to the Kosrae State Department of Health Services outbreak response team. Six serum specimens were submitted for reference laboratory testing by reverse transcription–polymerase chain reaction (RT-PCR) and anti-DENV IgM enzyme-linked immunosorbent assay (ELISA) to the Queensland Health Forensic and Scientific Services, Australia. Five of the six specimens were tested with RT-PCR using a novel dried-sera-on-filter-paper surveillance technique at the Institut Louis Malardé, French Polynesia (2). DENV-4 was detected by RT-PCR in one specimen at both laboratories, and four specimens had detectable anti-DENV IgM antibody, of which two were specific for DENV-4 (3). Thus, DENV infection was confirmed in five (83%) of six suspected dengue cases.

A modified WHO 2009 dengue case definition (4) was used to identify suspected dengue cases with fever plus at least two of the following: anorexia and nausea, rash, aches and pains (headache, eye pain, muscle pain, or joint pain), leukopenia (white blood cells <4,000/mL), or a warning sign (abdominal pain or tenderness, persistent vomiting, mucosal bleed or widespread petechiae, lethargy, restlessness, clinical fluid accumulation, or liver enlargement >2 cm). The case definition for an RDT-positive case was any suspected dengue case that tested NS1-positive or IgM-positive by RDT.

From September 26, 2012, to March 14, 2013, a total of 729 suspected dengue cases were identified at Kosrae State Hospital, with 242 (33.2%) patients admitted. One or more dengue warning signs were reported for 159 (21.8%) patients. Although detailed data on severe dengue cases are not available, poor peripheral perfusion or hemodynamic instability, as defined by the treating physician, was reported for 25 (3.4%) patients. No deaths were reported.

**FIGURE 1:**
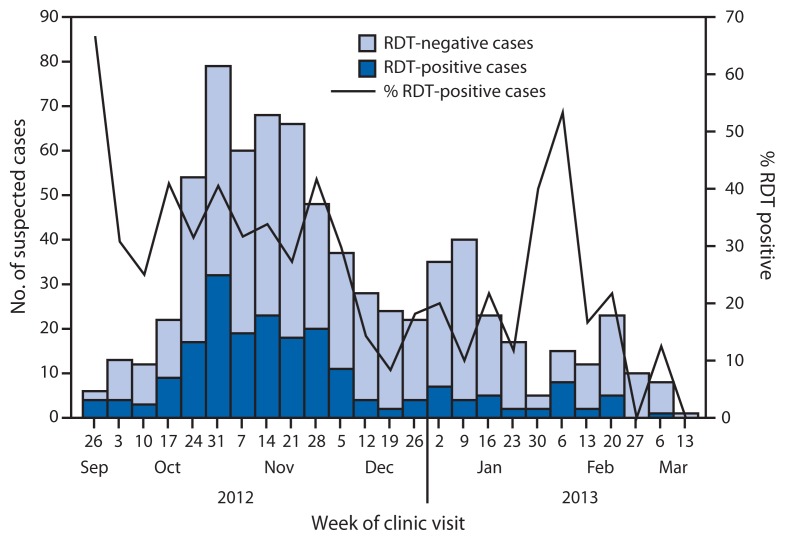
Number of suspected dengue cases (n = 728), by rapid diagnostic test (RDT) result and week of clinic visit — Kosrae State, Federated States of Micronesia, 2012–2013

Of 728 patients tested by RDT, 206 (28.3%) had positive results (Figure 1). Of these, 173 (84.0%) were NS1-positive and 41 (19.9%) were IgM anti-DENV-positive (eight patients [4%] were positive for both NS1 and IgM anti-DENV). The cumulative incidence during the 5.5 months was 110.0 suspected and 31.1 RDT-positive dengue cases per 1,000 population, respectively.

**FIGURE 2:**
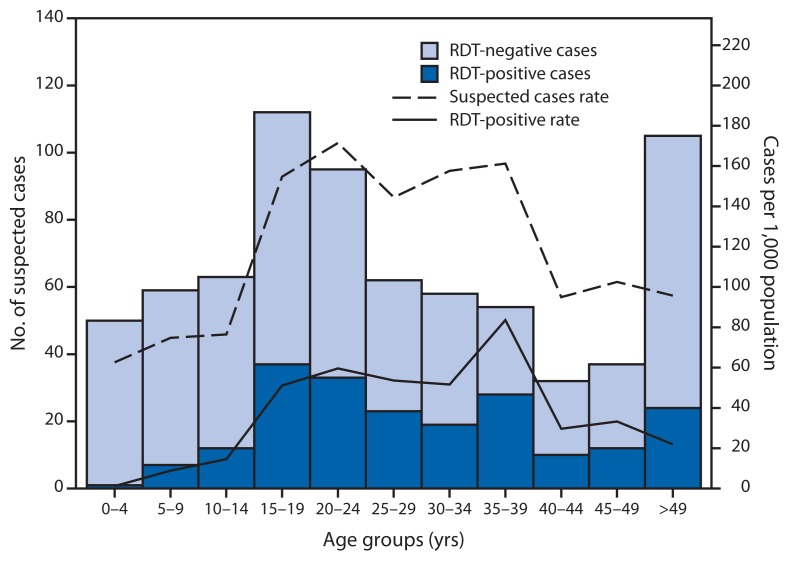
Number (n = 728) and rate^*^ of suspected dengue cases, by age group and rapid diagnostic test (RDT) result — Kosrae State, Federated States of Micronesia, 2012–2013 ^*^ Per 1,000 population

The persons most affected by the dengue outbreak were aged 15–39 years (Figure 2). The 20–24 year age group had the highest suspected dengue case rate at 171.5 per 1,000 population; the 35–39 year age group had the highest RDT-positive dengue case rate at 83.6 per 1,000 population. The least affected age group was 0–4 years (62.7 suspected and 1.3 RDT-positive cases per 1,000 population, respectively).

The most affected municipality was Lelu, where 42% (307 of 729) of Kasrae State’s suspected cases occurred, and the highest rates of suspected and RDT-positive dengue cases were recorded (142.1 and 39.8 per 1,000 population, respectively). Females accounted for 51.5% of suspected cases and 45.6% of RDT-positive cases.

The mosquito sampling survey identified *Aedes aegypti*, the primary global DENV vector, and *Aedes albopictus*, a secondary DENV vector. Of these two vectors, *Ae. albopictus* predominated, making up 94% of mosquito larval samples obtained in Lelu and 100% of those in the other three municipalities. In Lelu, used tires were the most common mosquito breeding sites, with 33% of tires having dengue vector larvae, followed by 55-gallon drums (11% with dengue vector larvae). Other breeding sites included coconut shells, buckets, flower pots, instant noodle cups, paper plates, and plastic containers (all <10%). Used tires also were the most common breeding site in Utwe municipality (80%), whereas pig feeding troughs were the most common sites in Tufansak municipality (22%).

## Public Health Response

The multiagency response team focused on coordination of outbreak response activities, conducting a needs analysis, optimizing clinical management, enhancing dengue surveillance, and augmenting hospital surge capacity when necessary. Communications messages were developed to encourage health-seeking behavior for persons with signs or symptoms of dengue, avoidance of mosquito bites, and eliminating mosquito breeding sites. Mosquito nets were provided for patients hospitalized with dengue and for pregnant women in the third trimester attending the antenatal clinic.

**FIGURE 3:**
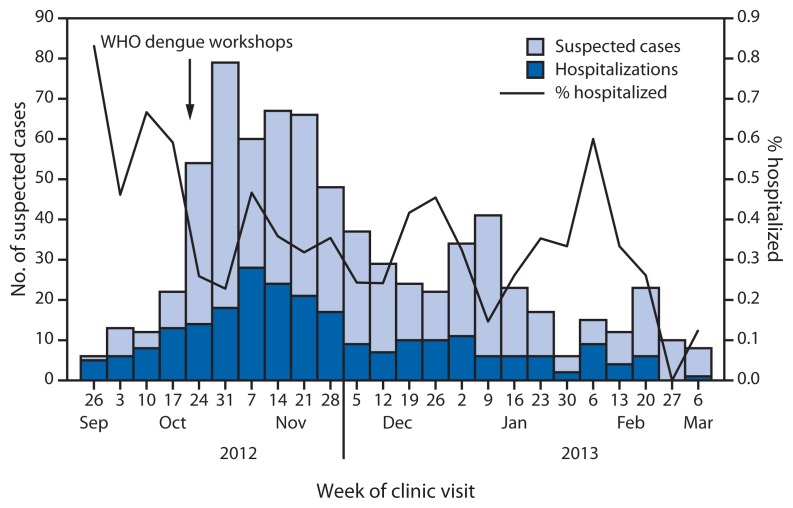
Number of suspected dengue cases (n = 728) and hospitalizations, by week of clinic visit — Kosrae State, Federated States of Micronesia, 2012–2013 **Abbreviation:** WHO = World Health Organization.

To maximize the quality of clinical care and the efficient use of resources, workshops for physicians and nurses on dengue clinical management were conducted, based on WHO guidelines (4). Training highlighted the importance of capillary leak as the primary pathophysiologic process that differentiated nonsevere from severe dengue, the importance of diligent hemodynamic monitoring, and the critical role of prompt and judicious administration of isotonic intravenous fluids to restore and maintain circulating blood volume. Emphasis was placed on maximizing available resources by triaging to ambulatory care the suspected dengue patients at low risk for developing severe disease (i.e., patients without warning signs) and admitting to the hospital those with warning signs of impending severe dengue or comorbidities that placed them at higher risk for severe disease. The dengue clinical management and WHO case-classification and triage workshops were conducted on October 25, 2012, and corresponded to a substantial decrease in the rate of hospitalizations, from 59.5% in the 2 weeks before the workshops to 24.6% in the 2 weeks after the workshops (Figure 3).

Enhanced dengue surveillance was established to ensure that all patients meeting the case definition were identified and tested by RDT, and that demographic, laboratory and clinical information were collected. Daily and weekly situational updates were circulated to local and external partners to provide an assessment of the outbreak trajectory and to monitor measures such as hospitalization rates and the percentage of RDT-positive suspected cases. During February 2013, the fifth month of the outbreak, the hospitalization rate and percentage of RDT-positive cases spiked substantially (Figures 1 and 3) because only the more serious dengue cases were being identified and tested. However, after clinicians were reminded of the need to test all cases meeting the suspected dengue case definition, the hospitalization rate and proportion of RDT-positive cases returned to baseline.

### Editorial Note

Dengue is caused by four closely related but distinct flaviviruses, DENV serotypes 1–4, which are transmitted by *Aedes* species mosquitoes. Dengue signs and symptoms include fever, aches and pains, nausea, rash, and mild bleeding. A small proportion of patients experience severe dengue, characterized by plasma leakage resulting in shock, respiratory distress secondary to ascites or pleural effusions, major hemorrhage, or serious organ impairment. Although there is no specific treatment for dengue, close monitoring of intravascular volume and prompt intravenous administration of isotonic crystalloids and/or colloids can be life-saving (4).

This report describes a DENV-4 outbreak in Kosrae State, FSM, in which >10% of the population received a diagnosis of suspected dengue and nearly 4% were hospitalized. Although slightly less than 30% of suspected dengue cases tested positive with a dengue RDT, this does not represent the true DENV infection rate, because the sensitivities of dengue RDTs are lower than diagnostic testing performed with immunoassays (5). In a previous DENV-4 outbreak in the Pacific, the sensitivity of the RDT used during this outbreak when compared with a combination of RT-PCR and anti-DENV IgM capture ELISA was found to be 66% (Dengue Branch, CDC, unpublished data, 2011).

DENV infection produces serotype-specific immunity; therefore, the preponderance of patients aged 15–40 years in this outbreak suggests that DENV-4 has not circulated in Kosrae for at least several decades, and the large susceptible population likely contributed to the magnitude of the outbreak. A possible explanation for the low rate of dengue cases in persons aged <15 years is that DENV-4 has been shown to be largely asymptomatic or to cause mild disease during primary DENV infection, whereas clinically apparent disease is more common during secondary infection with a different DENV serotype (6). This suggests that most of the clinically apparent cases seen during this outbreak might have been secondary DENV infections in persons who had experienced primary infection ≥15 years ago, consistent with exposure during the 1998 DENV-2 outbreak in Kosrae (1).

The epidemiology of dengue in the Pacific Islands is both unique and heterogeneous. Some outbreaks occur on very small, isolated islands and atolls with susceptible populations and highly efficient mosquito vectors leading to explosive but short-lived outbreaks, often burning out within weeks to several months. The outbreak in Kosrae is an example of this type of outbreak. Conversely, outbreaks on the geographically larger Pacific islands with larger and more widely distributed populations often result in prolonged circulation of a single serotype, but rarely for more than 4–5 years (7). Unlike most dengue-endemic regions, cocirculation of multiple DENV serotypes is unusual in the small Pacific islands (8). The present dengue outbreak likely was caused by a single serotype, but because of the small number of serotype-specific laboratory-confirmed cases, this cannot be confirmed.

What is already known on this topic?

Dengue outbreaks occur sporadically in many of the Pacific Island countries and territories. Over the past 4 decades, short-lived and often explosive dengue outbreaks have been reported. Dengue has not been reported in Kosrae State, Federated States of Micronesia, since 1998.

What is added by this report?

A dengue outbreak occurred in Kosrae State during September 2012–March 2013, in which 11% and 4% of the population met the case definition for suspected dengue and were hospitalized, respectively. Notable consequences of the outbreak included a substantial drain on a range of medical and public health services, including outpatient, inpatient and laboratory services, which resulted in redirection of limited human and material resources from other important medical and public health activities.

What are the implications for public health practice?

Because the health consequences can be substantial in these resource-limited settings, Pacific Island nations might consider planning for future dengue outbreaks by enhancing surveillance activities, ensuring laboratory testing capacity, and developing and testing outbreak response contingency plans.

Multiple dengue outbreaks have occurred in the Pacific over the past decade, affecting nearly all countries and territories. Periodic dengue outbreaks are expected to continue to occur in the Pacific. Surveillance for acute febrile illnesses could be strengthened in areas at risk for dengue to promptly identify outbreaks. As demonstrated during this outbreak, the public health impact of dengue in these resource-limited settings can be substantial, both in terms of morbidity and redirecting human and material resources away from other health priorities. Public health preparedness planners might consider including dengue among their priority diseases to ensure adequate capacity and resources to recognize and respond to the disease. Hospitalization of stable patients at low risk for developing severe dengue overloads inpatient services and expends valuable resources, but is common during outbreaks unless clinicians are trained in dengue case classification and management. As this outbreak demonstrated, timely and appropriate WHO dengue case classification and triage workshops can substantially reduce unnecessary hospitalization rates, although ongoing attention to key metrics (e.g., hospitalization or percentage of RDT-positive cases) is important to monitor application of triage criteria and case-identification protocols.

This outbreak highlights the multidisciplinary nature of the public health response required to manage a dengue outbreak. One area of particular importance is assuring high quality clinical management. This is often overlooked, despite reliable evidence that proper management can substantially reduce mortality from severe dengue (9). Public health messages during a dengue outbreak should recommend that residents and visitors 1) seek care for dengue-like illness; 2) eliminate mosquito breeding sites by covering, emptying, or disposing of water containers (e.g., water cisterns, discarded tires and refuse, and flower pots); and 3) protect themselves from being bitten by the predominantly day-biting *Aedes* species mosquitoes by using insect repellent, wearing insecticide-impregnated clothing, and assuring that intact mosquito screens are in place on doors and windows. Additional information on dengue is available from WHO at http://www.who.int/topics/dengue/en and from CDC at http://www.cdc.gov/dengue.
